# Advanced Immune Cell Profiling by Multiparameter Flow Cytometry in Humanized Patient-Derived Tumor Mice

**DOI:** 10.3390/cancers14092214

**Published:** 2022-04-28

**Authors:** Christina Bruss, Kerstin Kellner, Olaf Ortmann, Stephan Seitz, Gero Brockhoff, James A. Hutchinson, Anja Kathrin Wege

**Affiliations:** 1 Department of Gynecology and Obstetrics, University Medical Center Regensburg, 93053 Regensburg, Germany; christina.bruss@ukr.de (C.B.); kerstin.kellner@ukr.de (K.K.); oortmann@csj.de (O.O.); sseitz@csj.de (S.S.); gero.brockhoff@ukr.de (G.B.); 2 Department of Surgery, University Hospital Regensburg, 93053 Regensburg, Germany; james.hutchinson@ukr.de

**Keywords:** humanized tumor mice (HTM), humanized patient-derived xenograft (hPDX), breast cancer, hematopoietic stem cells (HSC), multicolor flow cytometry, immunotherapy

## Abstract

**Simple Summary:**

Immunotherapies have revolutionized the field of oncology and have been approved to treat cancer. Despite progress in immunotherapy, many challenges remain, including the identification (i) of predictive markers for treatment response or (ii) of beneficial T cell subsets involved in tumor elimination. “Humanized” mice are a promising translational model for studying the human immune system in the context of immuno-oncology research. Here, multicolor flow cytometry was applied to characterize immune cell subsets in the spleen of humanized mice transplanted with patient-derived breast cancer tissues. This multicolor immune cell setup will help to identify promising therapeutic approaches or predictive immune cell subsets in the future using humanized tumor mice.

**Abstract:**

“Humanized” mice have been widely used for the characterization of human cancer progression and as a powerful preclinical model. Standardization of multicolor phenotyping could help to identify immune cell patterns involved in checkpoint-related complications. Therefore, we applied established protocols for immune cell profiling to our humanized Patient-Derived Xenograft (hPDX) model. hPDX are characterized by the co-existence of a human immune system and a patient-derived tumor transplant. These mice possess a human-like immune system after CD34^+^ stem cell transplantation while the reconstitution level of the immune system was not related to the quantity of transplanted CD34^+^ cells. Contamination ≤ 1.2% by CD3^+^ cells in the hematopoietic stem cell (HSC) transplant did not trigger abnormal T cell maturation. Different B and T cell differentiation stages were identified, as well as regulatory T cells (Tregs) and exhausted T cells that expressed TIGIT, PD-1, or KLRG1. Overall, the application of standardized protocols for the characterization of immune cells using flow cytometry will contribute to a better understanding of immune-oncologic processes.

## 1. Introduction

Immunotherapies have extended the arsenal for tumor treatments, significantly contributing to tumor regression in cancer patients suffering from various malignancies, including melanoma, renal cell carcinoma, lung and urothelial cancer, as well as leukemia [1,2,3]. They have already been approved to treat many types of cancer and frequently trigger an efficient immunological tumor defense [4,5]. However, there are patients who do not benefit from the currently available immunotherapies. The immunological, environmental and cellular context before and during immunotherapies is incompletely explored and an individual prediction for treatment response is rather uncertain. Some patients develop immune-related adverse events or even experience rapid tumor progression, a so-called hyper-progressive disease [6,7,8]. In order to advance and improve these immunotherapeutic approaches, revised and more human-relevant preclinical models are crucial. Classical in vitro and in vivo studies in biomedical research have limitations because conditions in cell culture experiments and classical mouse models insufficiently mimic the conditions in the human body [9,10].

So-called humanized mice represent a promising model for studying the human immune system, optimizing the efficacy of applied therapies and ruling-out treatment-related toxicity. Numerous recent studies revealed the advantages of using humanized mice in the context of immune-oncologic research. The benefit of using these mice for cancer immunotherapy studies consists of three elements: (1) immunodeficient host mice bear (2) a human immune system and (3) human tumor cells at the same time. Therefore, humanized mice allow medical research and drug discovery in a more physiologically human-relevant setting. In particular, these models enable the analysis of the mutual interaction between the human immune system and human tumor growth, which reveals patient-relevant effects and, thus, enhances the clinical translation for treating patients with cancer.

The term “humanization” refers to the maturation and presence of a human immune system upon transplantation of hematopoietic stem cells (HSC) or peripheral blood mononuclear cells (PBMCs) into immunodeficient mice. Accordingly, these mice reflect human-like conditions with regard to the immunological context. A number of methods for humanization have been described in the literature [11]. More specifically, for the establishment of a functional human immune system, either PBMCs or CD34^+^ HSC can be used, which are transplanted into immunodeficient mice. The advantage of using CD34^+^ HSC, mainly isolated from umbilical cord blood after live births, is the differentiation of HSC into all major immune cell types resulting in a human-like immune system in a mouse without causing graft versus host diseases (GvHD). After removing mouse progenitor cells to create space in the bone marrow niche by sublethal irradiation, transplantation is performed by injecting HSC either into newborn mice, intravenously or intrahepatically, or into adult mice, typically by intravenous tail vein injection [11].

Depending on the research objective, it is critical to choose the right immunodeficient animal model [12,13]. NOD-*scid* IL2Rgamma^null^ (NSG) mice have a Non-Obese Diabetic (NOD) background resulting in the absence of circulating hemolytic complement [14] and diminished functions of natural killer (NK) cells [15], macrophages [16], and dendritic cells [17]. Due to the spontaneous Prkdc^scid^ mutation leading to defects in V(D)J recombination [18], NSG mice are characterized by the absence of functionally mature T and B cells. Moreover, the deficiency in the interleukin 2 receptor gamma chain (Il2rg) impedes the signaling of different interleukins (IL), namely IL-2, IL-4, IL-7, IL-9, IL-15, and IL-21 [19], contributing to a blockade of NK cell, T and B cell maturation.

To advance novel therapeutics in cancer research, humanized mice are being used as a translational model constituting a validated, powerful tool for preclinical investigation shown by several studies [20,21,22,23,24,25,26,27,28,29,30]. Especially in this context, experiments with humanized mice could contribute to recapitulating the interactions between immune components and tumors of human origin, expanding the knowledge in immuno-oncology translational research for better immuno-therapeutic drug development [29,30]. Even long-term efficacy of immunotherapies or biomarkers can be evaluated as persistence of human immune cells is shown in mice even after 11 months post CD34^+^ HSC transplantation [29]. However, standardized protocols for the humanization of mice and the subsequent analysis are indispensable [31]. An initiative called “Minimal information for standardization of humanized mouse models” (MISHUM) was built recently, to enable standardization and reproducibility of this model system [32].

Here, we applied a well-defined protocol for immune cell profiling [33] to the Humanized Patient-Derived Xenograft (hPDX) model. Immunophenotyping using flow cytometry has become the method of choice for the identification and advanced classification of immune cells. A flow cytometric approach facilitates the quantitative evaluation of an immunological activity on a single cell level. Therefore, we examined several flow cytometry panels that were already used to identify checkpoint-related complications in patients [34] to reliably quantify different immune cell populations in spleens of tumor-bearing humanized mice. Various B and T cell maturation and differentiation stages were identified. The heterogeneity of the B cell compartment known from the human body was also found in hPDX mice. A large portion of the T cells were assigned to the more experienced memory subsets. Furthermore, we detected regulatory T cells (Treg) and exhausted T cell subsets that expressed diverse markers, including TIGIT, PD-1 and KLRG1. Moreover, as CD4 and CD8 T cells were characterized by a human-like T cell receptor (TCR) repertoire, hPDX mice represent a powerful model for studies to optimize adoptive cell transfer. The immune cell composition in hPDX mice, analyzed by multicolor flow cytometry, was similar to the known profile in the human body. According to the principle from bench to bedside, we here demonstrate that the use of hPDX mice for the evaluation of immunotherapies is beneficial in paving the way towards successful clinical trials, in limiting and reducing severe unwanted side effects during the period of preclinical evaluation, and finally, in efficiently translating promising approaches to clinical applications.

## 2. Materials and Methods

### 2.1. The Breast Cancer Tumor Tissue

All patient-derived tissues samples were collected based on written consent and based on the permission of the Ethics Committee of the University of Regensburg. Breast cancer patients underwent surgery at the Department of Gynecology (University of Regensburg) and subsequently, tumor material was removed under sterile conditions. The tumor was incubated in pre-warmed medium (DMEM/F12, 1% HEPES, 1% Pen/Strep, 1% Amphotericin B) and minced into fragments of 2 mm × 2 mm. The tumor fragments were either transplanted subsequently into NSG mice or cryopreserved in liquid nitrogen.

### 2.2. Isolation of Human CD34^+^ Stem Cells from Umbilical Cord Blood

CD34^+^ hematopoietic stem cells (HSC) were isolated from the umbilical cord blood based on the procedure described the first time in 2011 [35]. Briefly, umbilical cord blood was collected postpartum in appropriate blood bags (Macopharma, Langen, Germany). Mononuclear cells (MNCs) were separated from cord blood by Pancoll density gradient centrifugation (PAN Biotech GmbH, Aidenbach, Germany) and washed twice in EDTA-PBS solution. CD34^+^ cell isolation was performed using immunomagnetic beads (Miltenyi Biotech, Bergisch Gladbach, Germany) according to the manufacturer’s instructions. If the cells were not used immediately after separation, the CD34^+^ cell fractions were cryopreserved in medium containing 45% FCS and 10% DMSO until further use.

### 2.3. Generation of Humanized Patient-Derived Tumor Mice (hPDX)

NOD.Cg-*Prkdc^scid^ Il2rg^tm1Wjl^*/SzJ (NSG) mice were obtained from Jackson Laboratories and housed and bred in a specialized pathogen-free facility at the University of Regensburg. Humanized tumor mice were generated as previously described [36]. In brief, newborn animals were irradiated with 1 Gy during the first 48 h of life span. After a resting phase of 3 h, mice were transplanted intrahepatically with 0.06–0.16 × 10^6^ human CD34^+^ cells isolated from umbilical cord blood. Reconstitution of the human immune system was analyzed 8–9 weeks after HSC transplantation (HSCT) by flow cytometry by blood collection over the lateral saphenous vein. Subsequently, mice were anaesthetized with midazolam (5 mg/kg), fentanyl (0.05 mg/kg) and medetomidine (0.5 mg/kg) i.p. and tumor fragments of four different patients were transplanted into the inguinal right fat pad together with 50 µL of matrigel (R&D Systems, Inc., Minneapolis, MN, USA). Anesthesia was antagonized using flumazenil (0.5 mg/kg), atipamezol (2.5 mg/kg) and naloxon (1.2 mg/kg).

### 2.4. Ethic Statements

The animal work was approved by the local veterinary authorities of the district government based on the European guidelines and national regulations of the German Animal Protection Act (permission no. 54-2532-1-16/14, 55.2 DMS-2532-2-422, and RUF 55.2.2-2532.2-803). Cord blood and patient-derived tumor samples were taken with approval from the Ethics Committee of the University of Regensburg (permission no. 14-101-0063, 17-527-101 and 18-1039-101). All patients included in the study provided written informed consent.

### 2.5. Flow Cytometry

The characterization of human immune cells was performed by flow cytometry using a FACSCanto-II (BD Biosciences, San Jose, CA, USA), which was run by Diva software (Ver. 7.0, BD Biosciences, San Jose, CA, USA) or a Navios cytometer (Beckman Coulter, Brea, CA, USA) with the Cytometry List Mode Data Acquisition and Analysis Software (Beckman Coulter, Brea, CA, USA).

The following anti-human antibodies (clones are given in brackets) were used for the analyses of the reconstitution in peripheral blood and in the CD34 isolates: αCD34-Pe (581, Biolegend), αCD3-FITC (UCHT1, BD Biosciences), αCD19-PE (HIB19, BD Biosciences), αCD33-PerCP-Cy5.5 (WM53, Biolegend), αCD45-APC (HI30, BD Biosciences).

To obtain single cell suspension of spleens for immune profiling, spleens were dissociated by passing the cells through a 40 μm cell strainer (BD Bioscience), eluated in PBS and centrifuged at 300× *g*. Samples were prepared using the DuraClone protocols according to the manufacturer’s protocol (Beckman Coulter, Brea, CA, USA). For detailed information see Table 1 and Kronenberg and colleagues [37]. The following anti-human antibodies (clones are given in brackets) were used and purchased from Beckman Coulter (Brea, CA, USA) if not otherwise stated: αCD16-FITC (3G8), αCD49b-FITC (P1E6-C5, Biolegend), αTCRγδ-FITC (IMMU510), αIgD-FITC (IA6-2), αCD45RA-FITC (2H4), αCD56-Pe (N901), αTIGIT-Pe (A15153G, Biolegend), αTCRαβ-Pe (IP26A), αCD21-Pe (BL13), αCD160-Pe (BY55), αCD19-ECD (J3-119), αCD27-ECD (1A4CD27), αCD8-ECD (B9.11), αCD14-PeCy7 (RMO52), αCD279-PeCy7 (PD1.3), αTCR Vδ1-PeCy7 (R9.12), αCD27-PeCy7 (1A4CD27), αTIGIT-PeCy7 (A15153G, Biolegend), αCCR7-PeCy7 (G043H7, Biolegend), αCD4-APC (13B8.2), αTIM3-APC (F38-2E2, Biolegend), αCD24-APC (ALB9), αCD127-APC (R34.34), αFoxP3-AF647 (259D), αCD8-APC-A700 (B9.11), αCD4-APC-A700 (13B8.2), αCD3-APC-A750 (UCHT1), αCD38-APC-A750 (LS198-4-3), αCD45-KrOrange (J33), αKLRG1-PerCP-Cy5.5 (SA231A2, Biolegend), αCD244-PeCy5.5 (C1.7), αCD25-PeCy5.5 (B1.49.9), αCD4-PB (13B8.2), αTCR Vδ2-PB (IMMU 389), αIgM-PB (SA-DA4), αHelios-PB (22F6). To exclude murine immune cells from our analysis, we added an anti-mouse αCD45 antibody in PerCP-Vio770 (30F11, Miltenyi), in PB (30-F11, Thermofisher Scientific), in APC-AF700 (30-F11, Thermofisher Scientific) or in Pe (30-F11, Miltenyi). The results were analyzed using the FlowJo software v10.8 (BD Biosciences, San Jose, CA, USA).

### 2.6. Statistical Analyses

All results are shown as mean. Correlation (linear regression) was performed using GraphPad Prism (Ver. 6, GraphPad Software, La Jolla, CA, USA).

## 3. Results

### 3.1. CD34^+^ Isolation from Umbilical Cord Blood and Cell Recovery after Cryopreservation

CD34 is the best-established marker for identification and isolation of HSC due to its unique expression on this cell type. Hence, it is chosen to enrich stem cells prior to transplantation and to determine the purity upon CD34 isolation. The amount of isolated CD34^+^ stem cells directly correlated with the volume of blood and separated MNCs (Figure S1A). On average, 19690 CD34^+^ cells were collected per ml blood (range 4950–44,720 cells mL^−1^). CD34^+^ stem cells were contaminated with only 0.33% CD3^+^ cells (range 0–1.2%) (Figure S1B). CD34^+^ cells were frozen with cryoprotectant solutions and stored in liquid nitrogen until further processing. The recovery rate was directly correlated to the cell concentration frozen (Figure S1C), 41.5% (range 20.5–73.0%) immediately after thawing (Figure S1D).

### 3.2. HSCT and Engraftment Success of the Human Immune System in Mice

**Figure 1:**
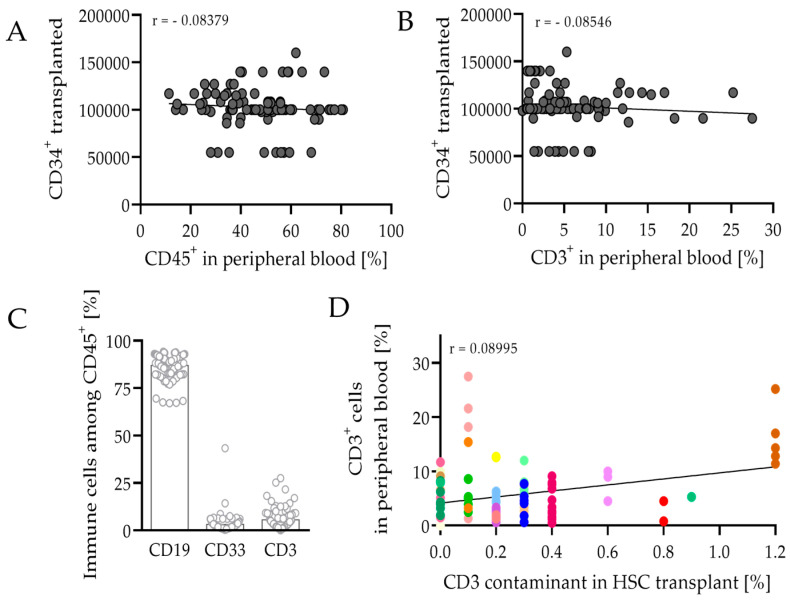
Engraftment success of the human immune system in mice 8–9 weeks after transplantation. (**A**) Frequency of reconstituted leukocytes (CD45^+^) and (B) T cells (CD3^+^) in peripheral blood of mice did not correlate with the transplanted number of CD34^+^ cells, analyzed by flow cytometry (mean). The linear regression is shown (Pearson’s correlation coefficient (**A**) r = −0.08379 and (**B**) r = −0.08546). (**C**) Distribution of reconstituted B cells (CD19^+^), myeloid cells (CD33^+^), and T cells (CD3^+^), among CD45^+^ leukocytes isolated from peripheral blood are depicted (mean). (**D**) Reconstitution of T cells in peripheral blood was determined by flow cytometry in comparison to T cell contaminant in the cord blood before transplantation. Each symbol represents one individual mouse. Individual colors display one particular transplant injected in several mice, respectively (*n* = 95).

Newborn pups were irradiated with 1 Gy whole-body irradiation during the first 48 h of their life span. After a resting phase, mice were transplanted intrahepatically with approximately 0.1 × 10^6^ human CD34^+^ cells (range 0.06–0.16 × 10^6^). Reconstitution of the human immune system was analyzed 8–9 weeks after HSCT by flow cytometry after blood collection from the lateral saphenous vein. Successful engraftment of humanization was considered when mice had more than 10% human CD45^+^ leukocytes in peripheral blood and was achieved in 89% of the 107 animals used. The quantity of the CD34^+^ cells transplanted did not correlate with the engraftment rate of human CD45^+^ leukocytes or CD3^+^ T cells (Figure 1A,B), which was also observed in other studies [38]. In transplanted mice, 47.4% (range 11.3–80.8%) of blood cells were CD45^+^. At this particular time, most of the cells belonged to the B cell compartment, as 86.8% (range 67.0–94.1%) were CD19^+^ among CD45^+^ leukocytes (Figure 1C). Of the cells, 3.2% (range 0.3–43.3%) were CD33^+^, a marker for the myeloid lineage. With a value of 5.5% (range 0.0–27.5%), T cells represented only a small proportion of CD45^+^ cells; however, this stage is seen as a turning point and the T cell concentration is expected to increase in peripheral blood after 8–9 weeks. At this particular time, higher CD3 portions are seen as critical, as mice tend to develop a GvHD. CD3 contamination in the CD34 fraction before transplantation did not contribute to the CD3 abundance (Figure 1D). Moreover, other factors in addition to the quality and quantity of the CD34 transplant play a pivotal role, as the recovered immune cells varied a lot between mice receiving the same transplant.

### 3.3. Immunophenotyping of Splenocytes in hPDX by Multiparametric Flow Cytometry

#### 3.3.1. Composition of Leukocytes Reveals Capability to Fight Cancer in hPDX

**Figure 2:**
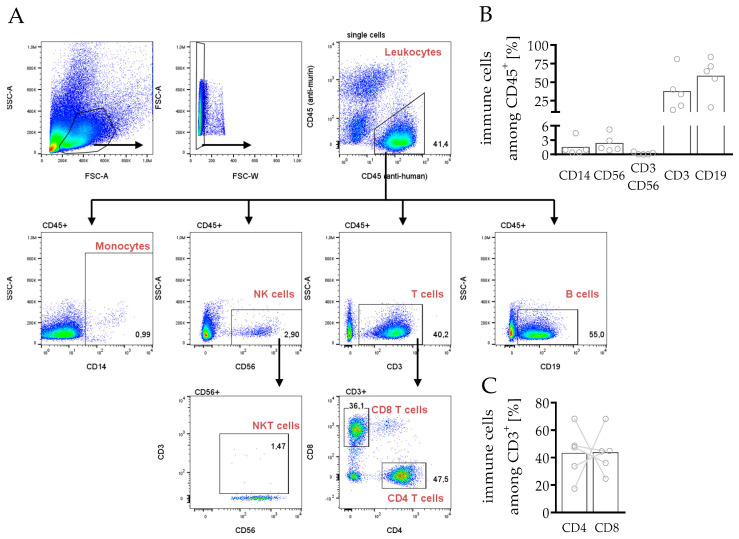
Immune profiling and gating strategy for phenotyping general leukocyte subpopulations in spleens of hPDXs. PDX tumors from breast cancer patients were transplanted orthotopically in humanized NSG mice. Spleens were harvested and processed to a single cell suspension. (**A**) Manual gating strategy for flow cytometry analysis of human splenocytes was as follows: singular (FSC-A FSC-W^low^) leukocytes (CD45^+^, human) were analyzed free from murine CD45^+^ cells regarding monocytes (SSC-A^low^ CD14^+^), NK cells (CD56^+^), NKT cells (CD56^+^ CD3^+^), T cells (CD3^+^), CD4 T cells (CD3^+^ CD4^+^), CD8 T cells (CD3^+^ CD8^+^) and B cells (CD19^+^). (**B**,**C**) Immune cell composition in spleens is shown. Each circle symbol represents one individual mouse (mean, *n* = 5).

In a first step, the main leukocyte subpopulations were analyzed to verify whether the human immune cell composition could be reproduced in the hPDX (Figure 2). For phenotyping human leukocyte subpopulations in the spleens, an anti-mouse CD45 to the pre-formulated DuraClone IM antibody cocktail from Beckman Coulter (Figure 2A) was added. T cells (37.3%) and B cells (58.2%) were the most pronounced of the present CD45^+^ immune cells, whereas monocytes (1.5%), NK cells (2.3%) and NKT cells (0.2%) were detectable only to a lower amount (Figure 2B). However, although the tendency of immune cell portions was similar in all mice, the composition varied between individual mice. In the human system, CD4 T cells are the major subset of T cells in peripheral blood, but in the spleen the amount of CD4 and CD8 T cells varied between the animals (Figure 2C). Importantly, the crucial, most important immune cells targeting tumor cells, in terms of NK and T cells, were present in hPDX.

#### 3.3.2. Differentiation and Maturation of B Cells in hPDX Are Comparable to the Known Human-like Heterogeneity

**Figure 3:**
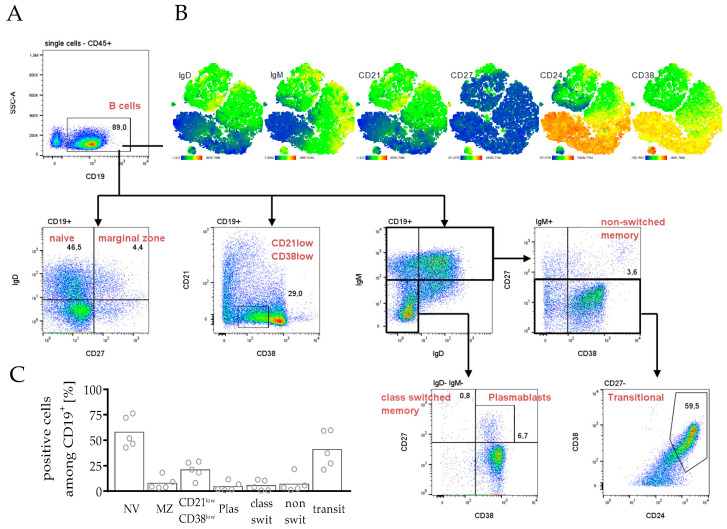
Immune profiling and gating strategy for phenotyping B cell subpopulations in spleens of hPDXs. PDX tumors from breast cancer patients were transplanted orthotopically in humanized NSG mice. Splenocytes were analyzed by flow cytometry. (**A**) Gating strategy for flow cytometry analysis of human splenocytes was as follows: singular (FSC-A FSC-W^low^, CD45^+^, human) B cells (CD19^+^), were analyzed free from murine CD45^+^ cells regarding the B cell subset markers CD21, CD24, CD38, CD27, IgD, and IgM. (**B**) Clustering of human B cells was calculated by t-SNE analysis, color-coded by the expression of different conventionally B cell markers as indicated. t-SNE map of one exemplary mouse is shown. (**C**) Analyses of B cell subpopulations of all hPDX are summarized: Naïve (NV) B cells (IgD^+^ CD27^−^), marginal zone (MZ) B cells (IgD^+^ CD27^+^), CD21 low CD38 low B cells, plasmablasts (Plas, IgD^−^ IgM^−^ CD27^+^ CD38^+^), class switched memory B cells (IgD^−^ IgM^−^ CD27^+^ CD38^−^), non- switched memory B cells (IgD^−/+^ IgM^+^ CD27^+^ CD38^+^), transitional B cells (transit, IgD^−/+^ IgM^+^ CD27^−^ CD38^−/+^ CD24^+^ CD38^+^). Each symbol represents one individual mouse (mean, *n* = 5).

As most leukocytes were assigned to B cells, the DuraClone antibody cocktail for the characterization of B cell subsets was applied to analyze splenocytes (Figure 3). The human B cell compartment displays a heterogeneous group, responsible for the humoral antibody response, carrying out antibody-independent functions such as antigen-presentation, modulation of T cells or the production of cytokines [39,40,41]. This heterogeneity was also found in the spleen of hPDX, when the core markers IgD, IgM, CD21, CD27, CD24 and CD38 (Figure 3A) were analyzed. t-SNE plot analysis revealed co-expression patterns for IgD, IgM, CD21, CD38 and partially CD24 expression displaying strong heterogeneity (Figure 3B). More than 50% of CD19^+^ B cells were naïve cells, mature but antigen-inexperienced B cells (IgD^+^ CD27^−^, Figure 3C). Moreover, additional precursor or differentiated B cell subtypes were found as memory B cells (switched, IgM^−^ IgD^−^ CD27^+^ CD38^−^ and non-switched IgM^+^ CD27^+^ CD38^+^), plasmablasts (IgM^−^ IgD^−^ CD27^+^ CD38^+^), or transitional B cells (IgM^+^ CD27^−^ CD24^+^ CD38^+^).

#### 3.3.3. Phenotyping and Characterization of CD4 and CD8 T Cells in hPDX

**Figure 4:**
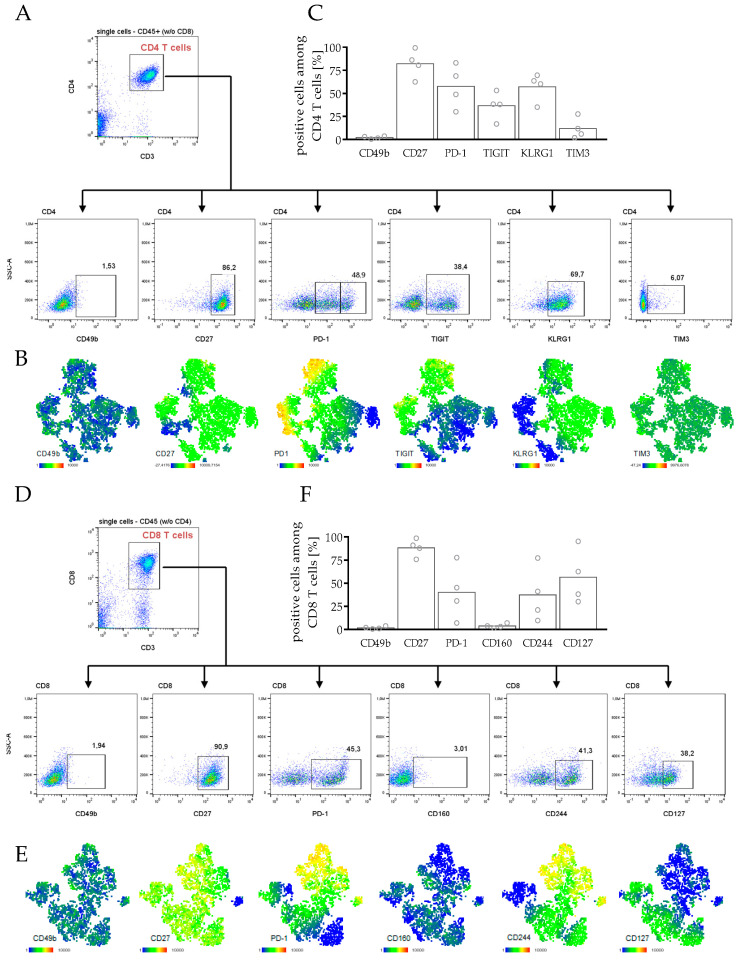
State of exhaustion in CD4 and CD8 T cells in spleens of hPDXs. PDX tumors from breast cancer patients were transplanted orthotopically in humanized NSG mice. Splenocytes were analyzed by flow cytometry. (**A**,**D**) Manual gating strategy for flow cytometry analysis in (**A**) CD4 and (**D**) CD8 T cells and is compared with t-SNE maps of (**B**) CD4 and (**E**) CD8 T cells, color-coded by the expression of different markers as indicated, to evaluate exhaustion phenotype by staining of CD49b, CD27, PD-1, TIGIT, KLRG1, TIM3 for CD4 T cells and CD49b, CD27, PD-1, CD160, CD244, CD127 for CD8 T cells. t-SNE maps of one exemplary mouse are shown. (**C**,**F**) Expression of exhaustion markers in (**C**) CD4 and (**F**) CD8 T cells in spleens of all analyzed hPDX are summarized. Each symbol represents one individual mouse (mean, *n* = 4).

**Figure 5:**
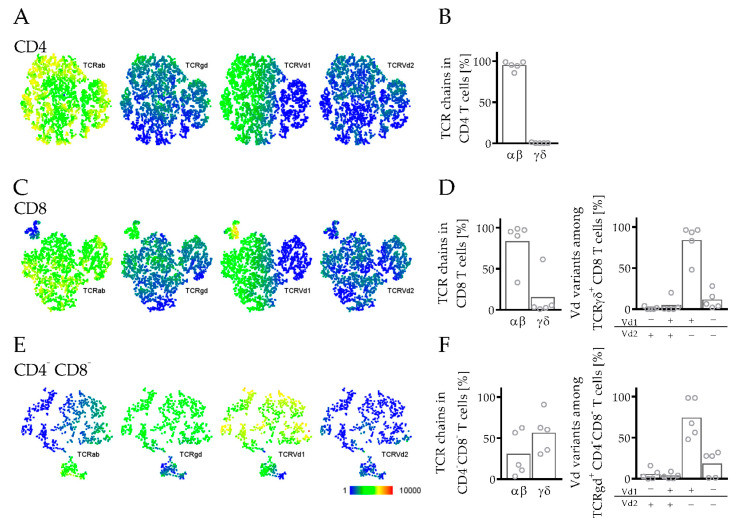
TCR repertoire in T cells in spleens of hPDX. PDX tumors from breast cancer patients were transplanted orthotopically in humanized NSG mice. Splenocytes were analyzed by flow cytometry. (**A**) CD4 T cells (CD3^+^ CD4^+^), (**C**) CD8 T cells (CD3^+^ CD8^+^) and (**E**) double-negative T cells (CD3^+^ CD4^−^ CD8^−^) were analyzed with t-SNE maps regarding the TCR chains TCRαβ (TCRab), TCRγδ (TCRgd), TCRvδ1 (TCRvd1) and TCRvδ2 (TCRvd2). (**B**,**D**,**F**) TCR repertoire analyzed by TCRαβ and TCRγδ (**B**) in CD4, (**D**) in CD8, and (**F**) in double-negative T cells and Vδ (Vd) variants in TCRγδ^+^ (**D**) CD8 and (**F**) double-negative T cells are shown. Each symbol represents one individual mouse (mean, *n* = 5).

**Figure 6:**
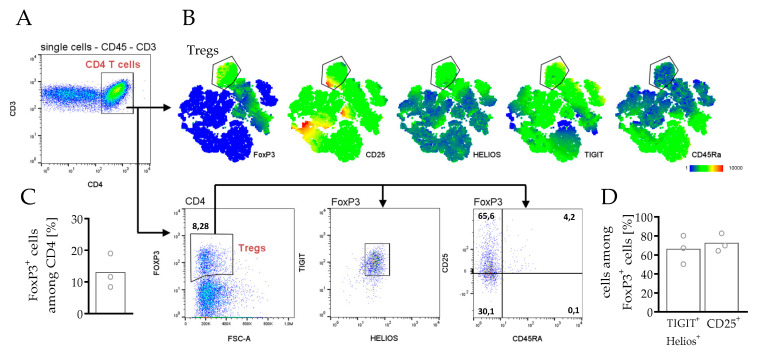
Phenotyping of CD4 regulatory T cells in spleens of HTMs. PDX tumors from breast cancer patients were transplanted orthotopically in humanized NSG mice. Splenocytes were analyzed by flow cytometry. (**A**) Manual gating strategy for flow cytometry analysis of human splenocytes is compared with (**B**) t-SNE maps of CD4 T cells, color-coded by the expression of different conventionally Treg markers as indicated. t-SNE map of one exemplary mouse is shown. (**C**) Quantity and (**D**) phenotyping of Tregs in spleens of all analyzed hPDX are indicated. Each symbol represents one individual mouse (mean, *n* = 3).

T cells are the key mediators of antitumor immunity. Therefore, we characterized CD4 and CD8 T cells regarding their subset composition (Figure S2), their exhaustion state (Figure 4), their TCR repertoire (Figure 5) and their regulatory potential (Figure 6) in hPDX mice. In contrast to the diverse numbers of CD4 and CD8 T cells (Figure 2C), the subset distribution was similar to the conditions known in the human body (Figure S2). The homing receptor CCR7 and CD45RA were used to distinguish between naïve (NV), central (CM) and effector memory (EM), and effector memory T cells expressing CD45RA (EMRA) (Figure S2A). Although high numbers of NV cells were found in spleens in the CD4 as well as in the CD8 compartment, a lot of cells were assigned to the more experienced CM and EM subsets (Figure S2B,C). Analysis of CD57 expression allowed the identification of terminally differentiated T cells with limited proliferative capacity (Figure S2B,C).

According to these results, CD4 as well as CD8 T cells were characterized by a high expression of markers associated with an exhausted phenotype (Figure 4). T cells entering an exhausted or dysfunctional state can limit the efficacy of immunotherapies. Therefore, we analyzed several exhaustion markers including TIGIT, KLRG1, and PD-1, which were upregulated in CD4 T cells (Figure 4A–C). However, CD27 expression was consistently high, and CD49b and TIM3 expression were not elevated. CD4 T cells expressing TIGIT also showed a strong PD-1 expression, but not vice-versa (Figure 4B). 

In the CD8 T cell compartment, in particular, an increased expression of PD-1, CD244 and partial of CD127 was observed, whereas the markers CD49b and CD160 were not upregulated (Figure 4D–F). PD-1 expression was concomitantly observed with CD244 expression but less with the other exhaustion markers (Figure 4E). Interestingly, in the CD4 T cells but not in the CD8 T cells, PD-1^+^ cells were separated into an intermediate and a high positive population. Whether progressive loss of effector functions such as IL-2, tumor necrosis factor (TNF), or interferon-γ (IFN-γ) production, which is usually coming along with T cell exhaustion, can be assigned to this T cell subpopulation, needs to be further elucidated. 

The TCR is a heterodimer composed by either an alpha (α) and beta (β) or a gamma (γ) and delta (δ) chain. In humans, most T cells belong to the αβ lineage, whereas only in 1–10% of T cells does the TCR consist of the γ and δ chain. In hPDX, especially, within the CD4 but also in CD8 T cells, most of the cells (>80%) were assigned to the αβ lineage (Figure 5A-D). In the CD8 compartment, a small portion of cells expressed the γδTCR, while most of them were Vδ1^+^ but Vδ2^−^ (Figure 5C,D). Vδs are variable segments of the γδ T cell receptor. Within the double negative T cell subset (CD3^+^ CD4^−^ CD8^−^), the major part belonged to the γδ T cells expressing mainly Vδ1^+^ (Figure 5E,F).

The presence of regulatory T cells in the tumor microenvironment is often associated with poor prognosis in patients. Therefore, spleen cells from hPDX were analyzed for the presence of regulatory T cells (Treg) characterized by the expression of the forkhead box protein 3 (FoxP3) (Figure 6). FoxP3^+^ cells made up to 13.0% in the CD4 T cell compartment (Figure 6C), expressing the characteristic Treg markers TIGIT and HELIOS, whereas not all Tregs were CD25^+^ (Figure 6B–D).

## 4. Discussion

Since the development of IL-2 receptor gamma chain knockout mice and combination with NOD*scid* mice, HSC transplantation has allowed the generation of a functional human immune system in mice, which results in the so-called humanized mice [12,13]. Since then, these mice have marked a new stage in biomedical research, e.g., in the field of infectious diseases, immunology, oncology, and others. Humanized mice facilitate an advanced evaluation of immune cell activity in a preclinical, but physiologically human-like setting.

NSG mice transplanted with CD34^+^ HSC develop the major immune cell populations within 12 weeks post-engraftment [11], even though some immune cells do not occur at the same frequency as they do in humans or do not completely mature towards absolute functionality [42]. Reconstituted immune cells varied between different mice receiving the same transplant (Figure 1). This phenomenon was already observed in humanized mice [38].

The development of the B cell compartment is still a subject of controversy. Human B cells are detectable in high frequencies in humanized mice but partly exhibit an immature phenotype [43]. However, humanized mouse models have shown human B cell activation and immunoglobulin production during infection [44,45] and in a variety of vaccine research studies [46,47]. Kuruvilla and colleagues demonstrated the production of dengue virus-specific antibodies with the ability to neutralize virus particles [44]. In humanized mice without human thymic tissue transplantation, human T cell differentiation and maturation occur on a murine major histocompatibility complex (MHC) background, and are therefore mostly H2-restricted. Due to the concomitant absence of CD4 helper cells and human-specific cytokine stimulation, class switch in B cells is reduced in HSC transplanted mice. Therefore, most of the human B cells show a CD24^int/hi^ CD38^hi^ immature phenotype [43]. However, co-transplantation of human tumor cells can trigger immune cell maturation and differentiation [48] and transitional B cells and plasmablasts can appear in hPDX (Figure 3). During the first nine weeks, humanized mice mainly generate human B cells (Figure 1), whereas human T cell numbers only start to increase at this stage. Probably due to an increase in mature T cells, which appear with the increasing age of the animals (Figure 2), B cells have the capacity to develop and to differentiate into mature subsets over time [49,50,51]. In addition, TLR ligand stimulation [52], the transgene expression of human cytokines [53,54,55,56], or HLA-DRA molecules delivered by lentiviruses [57] can further improve immunoglobulin production and class switch in these mice. The importance of B cells for an immunological tumor defense was highlighted recently with respect to breast cancer [58,59], which demonstrates the need to evaluate the impact of this cell type on tumor growth and restriction in more detail. Another striking concept is the utilization of humanized tumor-bearing mice to find and generate tumor-specific monoclonal antibodies [48,60], which is rather challenging due to the aforementioned complications of B cell maturation.

As already mentioned, mouse thymic epithelial cells express only murine MHC molecules, which possibly contributes to an impaired T cell differentiation [61]. T cell development was analyzed in several models and new mouse strains were generated expressing human MHC I and/or MHC II, which enables an improved maturation process of human T cells in the murine system [62,63,64,65]. In contrast to αβ T cells, γδ T cells develop MHC independently [66]. In the model presented here, progenitor cells differentiated into αβ and γδ T cells (Figure 5). γδ T cells were shown to have a potent antitumor activity, which caused special interest to translate γδ T cell activity into innovative immunotherapies [67], which can potentially be accomplished by the utilization of hPDX. Although studies demonstrated restricted T cell effector functions in different humanized mouse models [68,69], human T cells show the potential to secrete cytokines and have cytotoxic activity caused by the release of perforin or granzyme A and B [12,64,70].

In mice transplanted with liver and thymus fragments as well as mice based on HSC transplantation, the most presented subset in the blood was naïve cells, followed by memory and effector T cells, in the CD4 as well as in the CD8 compartment [71]. Likewise, we identified in hPDX all essential T cell subsets (Figure S2, [72]) and showed T cell maturation and activation from a naïve (CD45RA^+^ CD27^+^) towards a central memory (CD45RA^−^ CD27^+^) and an effector memory (CD45RA^−^ CD27^−^) phenotype in CD4 and CD8 T cells.

Due to the above-described maturation of a human immune system in humanized mice and hPDX mice, these models are particularly suitable for the preclinical in vivo evaluation of a variety of treatment strategies against cancer, above all immunotherapies. Antibodies targeting checkpoint molecules such as cytotoxic T-lymphocyte-associated protein 4 (CTLA-4) or programmed cell death protein-1 (PD-1) have been approved and are in clinical practice for the therapy of a variety of cancers [5]. However, due to significant insufficiencies in immunotherapies, there is a fundamental rationale for the evaluation of parameters involved in the response and resistance to exploring the impact of additional checkpoints, such as lymphocyte activation gene-3 (LAG-3), T cell immunoglobulin and mucin-domain containing-3 (TIM-3), T cell immunoglobulin and ITIM domain (TIGIT), and others [73]. As T cells in humanized mice express several checkpoint molecules, such as PD-1 or TIGIT, also shown in hPDX (Figure 4), studies with checkpoint inhibitors in humanized mice contribute to a better understanding of immunotherapeutic mechanisms taking place in humans [22,26,28,74,75,76]. For example, the following issues could be addressed by the use of HTM/hPDX in more detail: T cells can enter an exhausted or dysfunctional state, which is characterized by sustained expression of inhibitory receptors limiting the efficacy of immunotherapies [77]. Terminally exhausted T cells are known to account for the failure of immune therapies in cancer patients [78]. Moreover, PD-1 is often coordinately expressed with other co-inhibitory surface molecules, including for example LAG-3, CD244, CD160, among others [79], which were also identified here, in hPDX by multiparameter flow cytometry (Figure 4).

Another relevant field of immune cell regulation that affects tumor growth and cancer therapies concerns T cells with inhibitory activity. Tregs are specialized T cells that normally suppress the immune response, thereby maintaining homeostasis and self-tolerance in the human body. However, high numbers of Tregs found in several tumor entities are often associated with poor prognosis in cancer patients [80,81,82,83]. Therefore, currently, Treg targeting is combined with immunotherapy as, for example, checkpoint blockade is discussed, possibly to improve immunotherapeutic approaches [84]. Anti-PD-1 antibodies have been already demonstrated to decrease Treg cell populations in patients [85]. Humanized mice could contribute to this approach [86], as it was not only in the hPDX model that Tregs were present (Figure 6) but also in other studies with HSC-transplanted NSG mice [87,88,89].

Similar to the approach described here, flow cytometric protocols for immune cell profiling were already used to identify checkpoint-related complications in melanoma patients receiving combined PD-1/CTLA-4 blockade [34,90]. In another study, a flow cytometric assay was applied to analyze tumor and peripheral blood samples addressing the cellular “immunome” in patients with melanoma, breast cancer and glioblastoma [91]. Here, we show that those protocols can likewise be used in studies with hPDX or humanized tumor mice. These mouse models are particularly appropriate for translational research on immunotherapies under human-like conditions. A precise characterization of immune cell phenotypes, activation status and interactions between immune cells in tumors are critical to predicting patient outcomes. Moreover, a better understanding of the mechanisms underlying T cell exhaustion using humanized mice may lead to novel therapeutic interventions for patients and could reduce the limitation of the efficacy of immunotherapies. Lower response rates to checkpoint blockade were observed, for example in less immunogenic cancer [22,92], but using humanized mice will facilitate the evaluation of potentially complex mechanisms. However, a mouse model will not be able to mimic the situation in human patients completely but will further improve our understanding in the field of immuno-oncology. The experiments in this study add evidence to the potential and the margins of improvement in the employment of humanized mice and flow cytometry. hPDX models will help to expand knowledge in the field of immuno-oncology translational research [29,30], enable the identification of biomarkers for cancer progression or relapse, and will facilitate the development of combination strategies (e.g., irradiation and checkpoint blockade) in the future. The multicolor immune cell setup used in this study enables the identification of predictive immune cell subsets and provides a basis for the design of immune cell specific treatments in cancer patients.

## 5. Conclusions

The number of publications using humanized (tumor) mouse models is steadily increasing. The usefulness of those preclinical humanized mouse models for the investigation of human cancer growth and progression, and for the evaluation of treatment modalities is obvious. Standardized multicolor panels for flow cytometry will improve comparability of treatment studies. Multiparameter flow cytometry applied to humanized tumor mice enables (i) the identification of predictive immune cell subsets and (ii) the definition of novel immunotherapies with superior treatment efficiency. Overall, this study provides a protocol for the generation of humanized tumor mice and their immune cell profiling using up to 10-color panels for flow cytometry.

## Figures and Tables

**Table 1:** Anti-human and anti-mouse antibodies for flow cytometry.

	488 nm (Blue Laser)					638 nm (Red Laser)			405 nm (Violet Laser)	
	FITC	PE	ECD ^1^	PerCP-Vio770 PerCPCy5.5 PECy5.5	PeCy7	APC AF647	A700/ APC-A700	APC-A750	PB	KrOrange
**basic phenotyping**	CD16	CD56	CD19	CD45 ^2^ (mouse)	CD14	CD4 ^5^	CD8 ^7^	CD3	---	CD45 (human)
**T cell subsets**	CD45RA	---	CD27	CD45 ^2^ (mouse)	CCR7	CD4 ^5^	CD8 ^7^	CD3	CD57	CD45 (human)
**TCR**	TCRγδ	TCRαβ	---	CD45 ^2^ (mouse)	TCR Vδ1	CD4 ^5^	CD8 ^7^	CD3	TCR Vδ2	CD45 (human)
**B cells**	IgD	CD21	CD19	CD45 ^2^ (mouse)	CD27	CD24 ^5^	---	CD38	IgM	CD45 (human)
**exhausted CD4 T cells**	CD49b	TIGIT	CD27	KLRG1 ^3^	CD279	TIM3 ^5^	CD8 ^7^ + CD45 ^8^ (mouse)	CD3	CD4	CD45 (human)
**exhausted CD8 T cells**	CD49b	CD160	CD27	CD244 ^4^	CD279	CD127 ^5^	CD8 ^7^	CD3	CD4 + CD45 (mouse)	CD45 (human)
**TREGs**	CD45RA	CD45 (mouse)	CD8	CD25 ^3^	TIGIT	FoxP3 **^6^**	CD4 ^7^	CD3	Helios	CD45 (human)

^1^ R PE–Texas Red. Antibodies conjugated with: ^2^ PerCP-Vio770, ^3^ PeCy5.5, ^4^ PerCPCy5.5, ^5^ APC, ^6^ AF647, ^7^ A700, ^8^ APC-A700.

## Data Availability

The data presented in this study are available in this article and Supplementary Materials.

## Supplementary Materials

The following supporting information can be downloaded at: https://www.mdpi.com/article/10.3390/cancers14092214/s1, Figure S1: Isolation and yield of hematopoietic stem cells from umbilical cord blood. (A) Yield of stem cells (CD34^+^) from umbilical cord blood is depicted. Isolation was based on the MACS separation technique using immunomagnetic beads. CD34 expression was determined after separation by flow cytometry (*n* = 40). (B) Purity of CD34 transplant after separation was analyzed by flow cytometry and subsequent staining of CD3 (mean, *n* = 16). (C,D) Recovery of defrozen stem cells before transplantation is shown (C) in comparison to frozen cell concentration and (D) as mean (*n* = 24). Each symbol represents one individual CD34 stem cell transplant. Figure S2: Immune profiling and gating strategy for phenotyping of CD4 and CD8 T cell subsets in spleens of HTMs. PDX tumors from breast cancer patients were transplanted orthotopically in humanized NSG mice. Splenocytes were analyzed by flow cytometry. (A) Manual gating strategy for flow cytometry analysis of human splenocytes is shown, analyzing CD4, CD8 T cells and corresponding subsets, naïve (NV; CCR7^+^, CD45RA^+^), central memory (CM; CCR7^+^, CD45RA^−^), effector memory (EM; CCR7^−^, CD45RA^−^), effector memory, expressing CD45RA (EMRA; CCR7^−^, CD45RA^+^). (B,C) Immune cell composition and CD57 expression of CD4 and CD8 T cells in spleens is displayed. Each symbol represents one individual mouse (mean, *n* = 3).
