# Supplementary material for: Advanced Immune Cell Profiling by Multiparameter Flow Cytometry in Humanized Patient-Derived Tumor Mice
Source: Cancers (Basel). 2022 Apr 28;14(9):2214. doi: 10.3390/cancers14092214 (PMC9103756; doi:10.3390/cancers14092214)
Supplement: Supplementary file 1 [file cancers-14-02214-s001.zip › cancers-1699851-supplementary.pdf]

cancers

MDPI

Supplementary Materials

Advanced Immune Cell Profiling by Multiparameter Flow Cytometry in Humanized Patient-Derived Tumor Mice

Christina Bruss [1], Kerstin Kellner [1], Olaf Ortmann [1], Stephan Seitz [1], Gero Brockhoff [1], James A. Hutchinson [2] and Anja K. Wege [1,*]

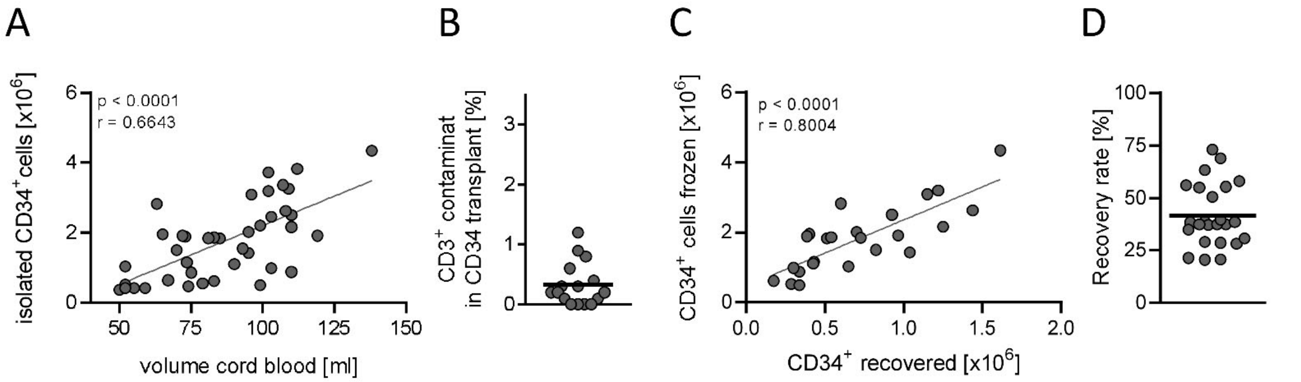

Figure S1. Isolation and yield of hematopoietic stem cells from umbilical cord blood. (A) Yield of stem cells ($CD34^+$) from umbilical cord blood is depicted. Isolation was based on the MACS separation technique using immunomagnetic beads. CD34 expression was determined after separation by flow cytometry (n=40). (B) Purity of CD34 transplant after separation was analyzed by flow cytometry and subsequent staining of CD3 (mean, n=16). (C, D) Recovery of defrozen stem cells before transplantation is shown (C) in comparison to frozen cell concentration and (D) as mean (n=24). Each symbol represents one individual CD34 stem cell transplant.

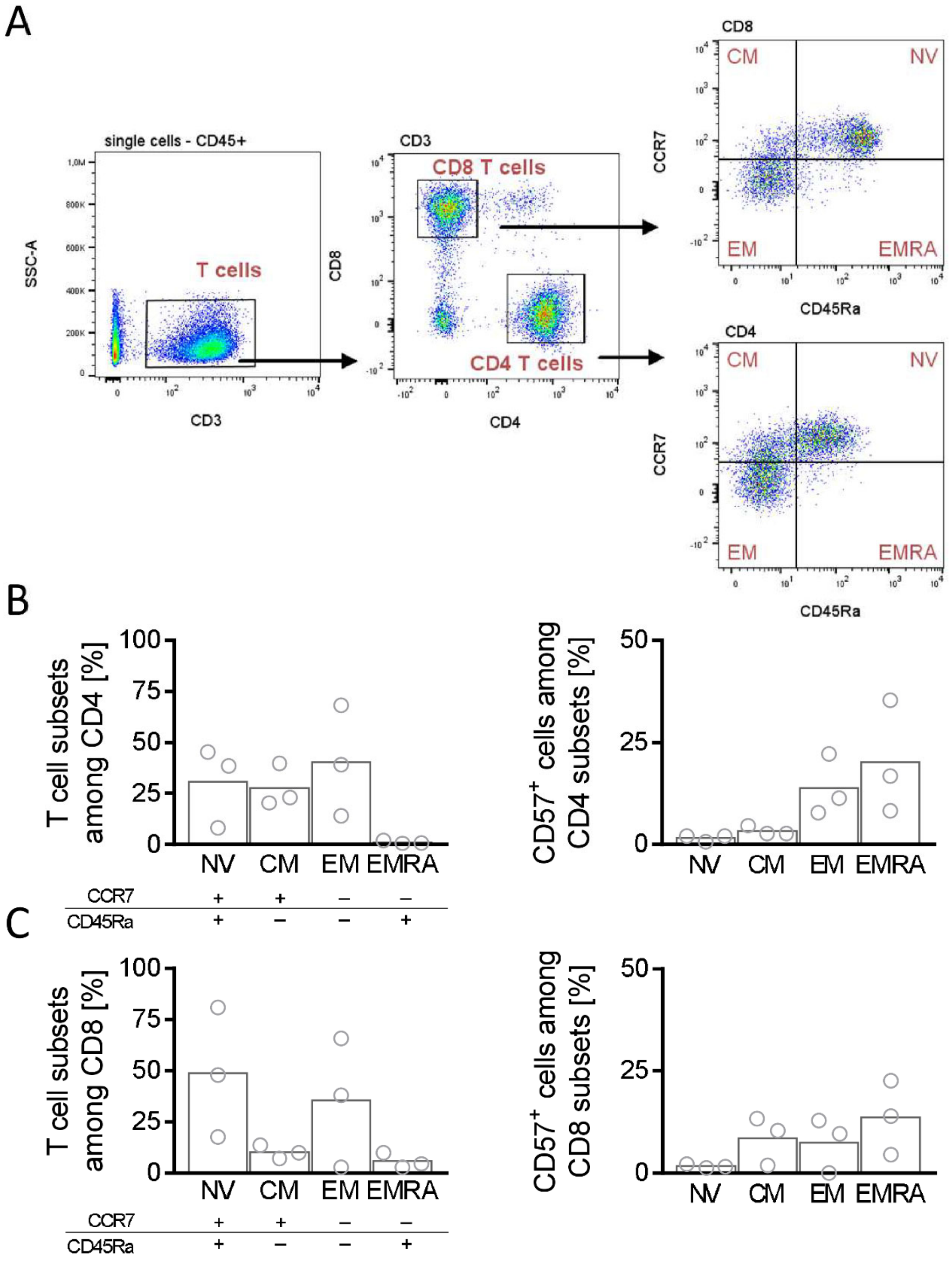

Figure S2. Immune profiling and gating strategy for phenotyping of CD4 and CD8 T cell subsets in spleens of HTMs. PDX tumors from breast cancer patients were transplanted orthotopically in humanized NSG mice. Splenocytes were analyzed by flow cytometry. (A) Manual gating strategy for flow cytometry analysis of human splenocytes is shown, analyzing CD4, CD8 T cells and corresponding subsets, naïve (NV; $CCR7^{+}$, $CD45RA^{+}$), central memory (CM; $CCR7^{+}$, $CD45RA^{-}$), effector memory (EM; $CCR7^{-}$, $CD45RA^{-}$), effector memory, expressing CD45RA (EMRA; $CCR7^{-}$, $CD45RA^{+}$). (B, C) Immune cell composition and CD57 expression of CD4 and CD8 T cells in spleens is displayed. Each symbol represents one individual mouse (mean, n=3).
